# Susceptibility of Mutant SOD1 to Form a Destabilized Monomer Predicts Cellular Aggregation and Toxicity but Not *In vitro* Aggregation Propensity

**DOI:** 10.3389/fnins.2016.00499

**Published:** 2016-11-04

**Authors:** Luke McAlary, J. Andrew Aquilina, Justin J. Yerbury

**Affiliations:** ^1^Lab 210, Illawarra Health and Medical Research InstituteWollongong, NSW, Australia; ^2^Science Medicine and Health Faculty, School of Biological Sciences, University of WollongongWollongong, NSW, Australia

**Keywords:** ALS, SOD1, protein misfolding, protein aggregation, mass spectrometry

## Abstract

Amyotrophic lateral sclerosis (ALS) is a fatal neurodegenerative disease characterized by the rapid and progressive degeneration of upper and lower motor neurons in the spinal cord, brain stem and motor cortex. The first gene linked to ALS was the gene encoding the free radical scavenging enzyme superoxide dismutase-1 (SOD1) that currently has over 180, mostly missense, ALS-associated mutations identified. SOD1-associated fALS patients show remarkably broad mean survival times (<1 year to ~17 years death post-diagnosis) that are mutation dependent. A hallmark of SOD1-associated ALS is the deposition of SOD1 into large insoluble aggregates in motor neurons. This is thought to be a consequence of mutation induced structural destabilization and/or oxidative damage leading to the misfolding and aggregation of SOD1 into a neurotoxic species. Here we aim to understand the relationship between SOD1 variant toxicity, structural stability, and aggregation propensity using a combination of cell culture and purified protein assays. Cell based assays indicated that aggregation of SOD1 variants correlate closely to cellular toxicity. However, the relationship between cellular toxicity and disease severity was less clear. We next utilized mass spectrometry to interrogate the structural consequences of metal loss and disulfide reduction on fALS-associated SOD1 variant structure. All variants showed evidence of unfolded, intermediate, and compact conformations, with SOD1^G37R^, SOD1^G93A^ and SOD1^V148G^ having the greatest abundance of intermediate and unfolded SOD1. SOD1^G37R^ was an informative outlier as it had a high propensity to unfold and form oligomeric aggregates, but it did not aggregate to the same extent as SOD1^G93A^ and SOD1^V148G^ in *in vitro* aggregation assays. Furthermore, seeding the aggregation of DTT/EDTA-treated SOD1^G37R^ with preformed SOD1^G93A^ fibrils elicited minimal aggregation response, suggesting that the arginine substitution at position-37 blocks the templating of SOD1 onto preformed fibrils. We propose that this difference may be explained by multiple strains of SOD1 aggregate and this may also help explain the slow disease progression observed in patients with SOD1^G37R^.

## Introduction

Amyotrophic lateral sclerosis (ALS) is a disease characterized by the progressive degeneration of both upper and lower motor neurons, ultimately resulting in death. The symptoms of ALS typically manifest in people aged 55–65 years, with younger patients rarely being identified (Gordon, 2011). Post-diagnosis, the average disease duration is approximately 3 years, although patients suffering different familial variants can have shorter and longer durations. Genetic studies of ALS patients have revealed that 90% of cases are sporadic (sALS) and the remaining 10% of cases are familial (fALS) (Renton et al., 2014). Several of the fALS associated genes (TDP-43, C9orf72, FUS, ALS2) have roles in the biogenesis and trafficking of RNA species, and another clear grouping are associated with protein degradation (CCNF, VCP, SQSTM1, UBQLN2, OPTN, TBK1); most are also linked to frontotemporal dementia (Cirulli et al., 2015; Peters et al., 2015; Williams et al., 2016). The first fALS linked gene discovered, and most studied, is the gene encoding superoxide dismutase-1 (SOD1) which is only associated with a pure motor neuron disease phenotype.

Superoxide dismutase-1 (SOD1) is a cytosolic 32 kDa homodimeric enzyme that catalyzes the dismutation of oxygen radicals to either molecular oxygen or hydrogen peroxide (McCord and Fridovich, 1969). Each subunit contains a catalytically active copper ion, a zinc ion, and an intramolecular disulfide bond. These post-translational modifications confer substantial stability to the native fold of SOD1, endowing it with resistance to heat denaturation and maintenance of catalytic activity in denaturing conditions (Senoo et al., 1988; Rodriguez et al., 2002). Zinc binding occurs spontaneously following synthesis and folding, whereas copper binding and disulfide bond formation are mediated by association with the copper-chaperone for SOD1 (Rae et al., 2000; Furukawa et al., 2004). Currently, there are known to be over 180 natural mutations that can occur within the SOD1 sequence that are associated with fALS [http://alsod.iop.kcl.ac.uk - (Abel et al., 2013)]. These mutations occur throughout the SOD1 sequence, and it is thought that all have the ability to destabilize the native fold, leading to an increased propensity to misfold and/or aggregate (Khare et al., 2006; Nordlund and Oliveberg, 2008; Byrstrom et al., 2010; Munch and Bertolotti, 2010). Owing to the growing evidence for prion-like propagation associated with SOD1 fALS (Ayers et al., 2014, 2016; Grad et al., 2014; Zeineddine et al., 2015), and the fact that a misfolded/aggregated wild-type SOD1 species has been found associated with sALS (Bosco et al., 2010), it has been suggested that there is a common pathogenic conformer or aggregate species that exists for all SOD1 variants, although it is yet to be elucidated.

Metal coordination and disulfide bond formation are important for stability of SOD1 tertiary and quaternary structures (Hayward et al., 2002; Tiwari and Hayward, 2003; Lynch et al., 2004; Svensson et al., 2010), and their absence has been shown to increase the aggregation propensity of SOD1 (Arnesano et al., 2004; Ding and Dokholyan, 2008). From this, it has been inferred that an immature/unfolded conformation(s) of SOD1 is responsible for aggregation. Investigation of these possible unfolded SOD1 conformations has been difficult due to the heterogeneity of non-native protein structures. High resolution analyses, such as X-ray crystallography, are impeded by the existence of disordered protein regions that can assume multiple conformations (Banci et al., 2009), and typical separation techniques, such as size exclusion chromatography (SEC) do not provide sufficient resolution to separate different conformations for downstream analysis (Hong et al., 2012), although use of small-angle X-ray scattering coupled with SEC can provide valuable insight into the structural plasticity of proteins in solution by separating different conformers and oligomeric states (Wright et al., 2011, 2016; Furukawa et al., 2015). Nuclear magnetic resonance (NMR) spectroscopy has been used to great extent on disordered SOD1, monitoring unfolding, refolding, and even identifying a zinc-deficient form of SOD1 proposed as a precursor to the toxic species (Luchinat et al., 2014; Szpryngiel et al., 2015). Although NMR analysis is an extremely useful tool in structural biology, it requires high concentrations of protein (0.1−3 mM), which can lead to undesirable artifacts, especially in the case of protein-protein interactions.

In comparison, native mass spectrometry (MS) is a powerful tool for the analysis of structurally heterogeneous protein samples as it allows for the separation of different protein species based upon mass, charge, and drift time when coupled to ion mobility (IM-MS) (Mehmood et al., 2015), allowing for the resolution of species in solution at variable concentrations. This study details the use of native MS to investigate the possible misfolded/unfolded conformations SOD1 can occupy when metal binding and disulfide bonding are disrupted, and the relationship of these species to SOD1 aggregation. We first determined if there was a correlation between isolated recombinant protein aggregation and cellular aggregation of different SOD1 variants, focusing on SOD1-EGFP fusion proteins. Next we attempted to assess the oligomeric state of our SOD1 variants under similar aggregation conditions, finding that SOD1 variants had differential mobility in both SEC and native PAGE. This was followed by native MS analysis to separate and analyse the variably destabilized SOD1 species. Our observations indicate that the oligomeric state of the SOD1 variants was primarily monomeric and that the monomer assumed 3 conformations based upon mass-to-charge distributions. The relative abundances of the conformations were dependent upon the SOD1 variant being analyzed and the formation of destabilized SOD1 monomer correlated with cellular aggregation. Lastly, the slow progressing SOD1^G37R^ mutant had a high proportion of unfolded monomer, high propensity to form oligomeric aggregates and insoluble aggregates, but could not be seeded by G93A aggregates in a plate-based aggregation assay, suggesting that it may exist in a different strain to other aggregation prone mutants.

## Methodology

### Expression and purification of recombinant SOD1

Plasmids encoding SOD1^WT^ and SOD1^G93A^ were a gift from the Oliveberg group (Stockholm, Sweden). Plasmids encoding SOD1^G37R^, SOD1^H46R^, SOD1^D90A^, and SOD1^V148G^ were designed in-house and generated by Genscript (New Jersey, USA). Protein expression and purification were performed according to (Lindberg et al., 2002) with slight modifications. Briefly, plasmids containing genes for the expression of SOD1 and yeast-CCS were transformed into chemically competent BL21 (DE3) *E. coli* using heat-shock. Following transformation expression cultures were induced using IPTG in the presence of copper and zinc. Following lysis and ammonium-sulfate precipitation, the expressed protein was purified using size exclusion chromatography (Hiload 16/60 Superdex 75 PG, GE USA) and anion exchange chromatography (HiTrap DEAE, GE USA). Purity was assessed by SDS-PAGE and mass spectrometry, with pure samples snap frozen and stored in 1 × PBS at −20°C. Misfolded/unfolded SOD1 was generated by incubating purified SOD1 variants at a concentration of 30 μM in 1 × PBS with 5 mM EDTA and 20 mM DTT at 37°C for 2 h. All protein concentrations were determined using a bicinchoninic acid assay.

### Isolated recombinant protein aggregation assays

Superoxide dismutase-1 (SOD1) variants were aggregated at a concentration of 30 μM (dimer) in 1 × PBS (pH 7.4) containing 20 mM DTT, 5 mM EDTA, with 10 μM thioflavin T (ThT). Plate-reader assays were performed on a POLARstar Omega (BMG labtech) in clear bottomed 384-well plates (Greiner), with a final well volume of 50 μl. Following addition of DTT/EDTA to wells containing SOD1 protein, the plate was incubated at 37°C for 30 min before being covered with an adhesive slip. Aggregation was induced with double-orbital shaking at 300 rpm for 330 s at the start of a 900 s cycle. ThT was excited at 440 nm and its fluorescence was measured at 490 nm. Seeded aggregation assays were carried out similarly, with the exception that the reaction mixture was 30 μM SOD1, 10 mM DTT, 1 mM EDTA, 10 μM ThT, in 1 × PBS (pH 7.4). Seeded assays also contained 0.3% (w/w) of total protein as seed from a previous aggregation assay. Analysis of aggregation kinetics was carried out as described by Cox et al. (2016).

### Native page analysis

Superoxide dismutase-1 (SOD1) variants at a concentration of 30 μM (10 μg total protein per well) were loaded into stain-free any kD gradient gels (Biorad, USA) in a tris-glycine buffer. Gels were electrophoresed at 100 V for 3 h at 4°C with stirring. Following electrophoresis, gels were imaged on a stain-free imager (Biorad, USA).

### Analytical gel-filtration and buffer exchange

Analytical gel-filtration chromatography was carried out in conjunction with buffer exchange of SOD1 variants into 200 mM NH_4_OAc (pH 6.8). SOD1 variants were concentrated using microfuge concentrators (Vivaspin 25 10 kDa MWCO, GE USA) to a concentration of ~450 μM prior to analytical gel filtration. Concentrated native and DTT/EDTA-treated SOD1 variants were loaded onto either a Superdex-75 10/300 column or Superdex-200 10/300 column (GE, USA) at a flow rate of 0.5 ml/min with the elution profile being measured at 280 nm, and 0.5 ml fractions being collected for immediate MS analysis. Fractions were placed on ice as they eluted.

### Mass spectrometry

Mass spectrometry analysis was performed using a SYNAPT G1 HDMS (Waters, UK) with parameters set according to previous work (McAlary et al., 2013). Briefly, SOD1 samples at 10 μM in 200 mM NH_4_ OAc were loaded into gold-coated borosilicate capillaries (made in-house) and subjected to nano-electrospray ionization. All spectra were externally calibrated using 10 mg/ml caesium-iodide in 50% n-propanol, and were processed using Masslynx 4.1. For determination of the abundances of the observed conformations MATLAB R2014b (Version 8.4) was used to fit Gaussians to the data.

### Secondary tissue culture

Mouse neuroblastoma/motor neuron hybrid cells (NSC-34) (Cashman et al., 1992) were cultured in Dulbecco's modified Eagles medium-F12 (DMEM-F12) (Invitrogen, Australia), supplemented with 10% (v/v) heat inactivated fetal bovine serum (FBS) (Bovogen Biologicals, Australia). In order to passage and plate cells, they were washed once with DMEM-F12 and treated with 0.25% trypsin, 0.02% EDTA (Sigma-Aldrich, Australia) to lift off the adherent cells. The cells were pelleted via centrifugation (500 × *g*, 5 min) and resuspended in pre-warmed DMEM-F12, supplemented with 10% FBS. Following washing, plates and chamber-slides were seeded at a confluency of 40% and cultured at 37°C in a humidified incubator with 5% atmospheric CO_2_ for 24 h prior to transfection.

### Transfection of NSC-34 cells

NSC-34 cells were transfected, 24 h post-plating into either multi-well plates or chamber-slides (Ibidi Germany), using Lipofectamine 3000 (Invitrogen, USA) according to the manufacturer's instructions. Lipofectamine 3000 reagent was added to pre-warmed DMEM-F12 to a concentration of 6% (v/v). P3000 reagent and plasmid DNA were added to a separate DMEM-F12 volume to concentrations of 4% (v/v) and 20 ng/μL, respectively. The DNA/lipofectamine complex was generated by mixing one volume of 6% (v/v) lipofectamine in DMEM-F12, with one volume of DNA/P3000 mix in DMEM-F12. The DNA/lipofectamine mixture was incubated for 30 min at room temperature, after which it was dispensed onto cultured cells. For cells that were co-transfected, the total amount of DNA was halved between plasmid constructs.

### Fluorescent imaging

All cell imaging was performed using a Leica TCS SP5 Confocal microscope (Leica, Germany). Cells were imaged live in 5% atmospheric CO_2_, with the incubation unit set to a temperature of 37°C. For all confocal experiments, the argon laser power was at 20%. All green-fluorescent protein (EGFP) tagged constructs were excited with 10% laser power at 488 nm and emission was analyzed from 507 to 550 nm. Images were acquired using a HCX PL APO 63.0 × 1.20 water immersion objective, using PMT detectors, with a pin-hole of 100 μm.

To quantify inclusion formation of SOD1-EGFP constructs, z-stacks of transfected cells were acquired. Cells were cultured into μSlide 8-well chamber slides (Ibidi, Germany) and transfected with c-terminal SOD1-EGFP constructs. Imaging was performed 48 h post-transfection. Z-stacks series were acquired at 512 × 512 pixels, laser scanning speed 400 Hz, line and frame averaging = 1, where each image was a 400-500 nm slice of the z-axis. Each Z-stack was systematically acquired in a new area of the well to avoid overlap. At least five z-stack series were acquired per well of a chamber slide, accounting for at least 100 transfected cells imaged per well. Z-stacks were processed into a single image using LAS-AF Lite software (Leica, Germany), where cells containing inclusions were determined by the presence of bright fluorescent puncta in the cytoplasmic region greater than 2 μm in diameter.

### Time resolved fluorescent imaging

To assess the toxicity of SOD1-EGFP constructs in NSC-34 cells over a time course, an IncuCyte® automated fluorescent microscope (Essen BioScience, USA) was used. NSC-34 cells were plated into 12-wells plates at a confluency of 60% and were transfected 24 h post-plating. Cells were dissociated 24 h post-transfection and plated into 96-well plates at a confluency of 20% in phenol-red-free DMEM-F12 supplemented with 10% FBS. At least 3 images were acquired per-well at 2 h time points for 68 h in both phase and green channels with the green channel acquisition time at 400 ms. The processing definition generated to analyse the images utilized top-hat back ground subtraction [radius = 100 μm, threshold = 0.5 general calibration units (GCU)], edge-splitting (edge sensitivity = 0), filters (minimum area = 175 μm^2^, minimum mean intensity = 1.2). The equations below describe data analysis and presentation.

First, the number of GFP positive cells at each time point (*GFP*_*tx*_) for each transfection was normalized to the intial value (*GFP*_*t*__0_) determined in the first scan after plating.

$$\begin{array}{l} \frac{GFP_{tx}}{GFP_{t0}}=NormalisedGFP_{tx} \end{array}$$

Then, the normalized values of the SOD1 mutants at each time point (*Normalised GFP*_*tx*_) were divided by the normalized *SOD*1^WT^ data at the same time points to determine the proportion of GFP positive cells relative to *SOD*1^*WT*^.

$$\begin{array}{l} \frac{NormalisedGFP_{tx}}{SOD1^{WT}GFP_{tx}}=SOD1^{mutant}GFPrelativetoSOD1^{WT} \end{array}$$

### FloIT assay by flow cytometry

The FloIT assay was performed as described in (Whiten et al., 2016). Briefly, NSC-34 cells were transiently transfected in a 24-well plate with SOD1-EGFP constructs as described above. Cell were harvested, washed twice and resuspended in 1 × PBS (0.5 mL/tube). A 150 μL aliquot was taken and used to analyse transfection efficiency. The remaining cells were pelleted (300 × *g*, 5 min) and placed on ice. Supernatant was removed from each sample and the cells were lysed with the addition of 0.5% Triton X-100 in 1 × PBS containing a 1:1000 dilution of RedDot2 immediately prior to flow cytometric analysis. Events were analyzed using a LSRFortessa X-20 Cell Analyzer (BD Biosciences) with the excitation set to 488 nm and the emission collected with a 525/50 band-pass filter for EGFP, and an excitation of 640 nm with the emission collected using a 670/30 nm band-pass filter for RedDot2. All parameters were set to log^10^ during acquisition from cell lysates, which were gated according to forward and side scatter. The forward scatter threshold was set to the minimum value (200 AU) to minimize the exclusion of small protein inclusions. Nuclei were identified based on RedDot2 fluorescence and forward scatter for removal from further analysis. The remaining particles were analyzed for presence of inclusions based on EGFP fluorescence, forward scatter, and comparison to lysates of NSC-34 cells expressing EGFP only. The number of inclusions in the population of particles was normalized to the number of nuclei, and reported as inclusions/100 transfected cells (*i*_*FloIT*_) according to the equation

$$\begin{array}{l} i_{FloIT}=100\left(\frac{n_{i}}{\gamma .n_{nuc}}\right) \end{array}$$

where n_i_ represents the number of inclusions acquired, *n*_*nuc*_ is the number of nuclei acquired, and γ is the transfection efficiency (expressed as a fraction).

## Results

### Cellular aggregation correlates with cell loss

**Figure 1:**
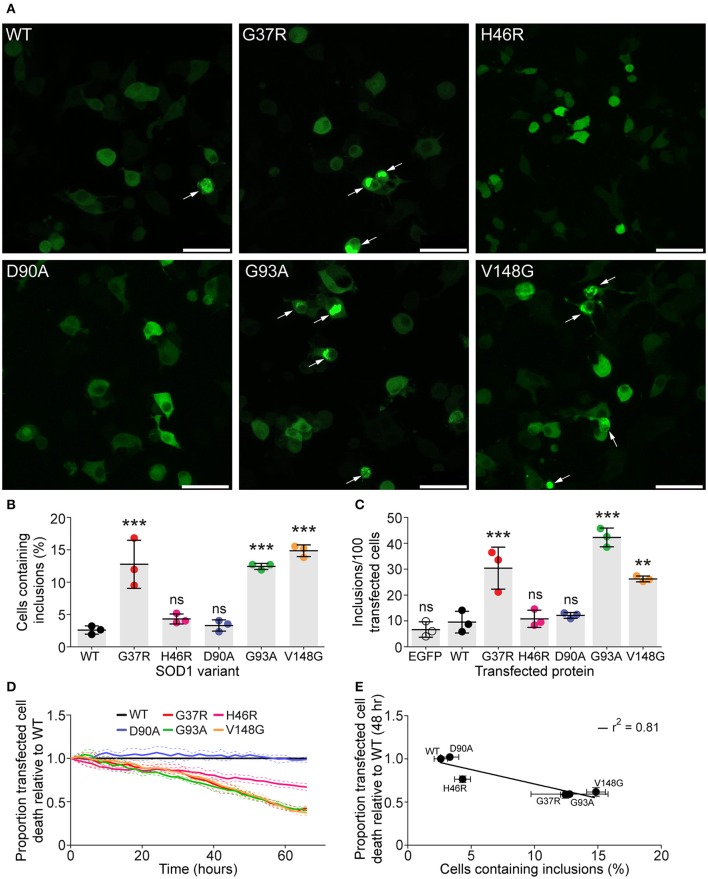
**SOD1 variants have differential aggregation propensities in NSC-34 cells**. NSC-34 cells were transiently transfected with SOD1-EGFP constructs and imaged 48 h following transfection to examine the formation of inclusions. **(A)** Representative images of NSC-34 cells transfected with different SOD1-EGFP variants with arrows marking cells with inclusions. Scale bar represents 50 μM. **(B)** Manual counting of inclusions from imaged cells showed that SOD1variants had differential aggregation propensities when expressed in NSC-34 cells. **(C)** FloIT analysis of transfected NSC-34 cells. Cells were lysed and the inclusions counted using flow cytometry 48 h post-transfection. **(D)** The toxicity of SOD1-EGFP constructs compared to SOD1^WT^ in NSC-34 cells. **(E)** Cell death is plotted against cellular aggregation propensity, there is a plausible correlation between aggregate load and cell death at 48 h post-transfection. Statistical significance was determined via a one-way ANOVA with a Tukey's multiple comparison post-test (^***^P <0.001, ^**^P <0.01, ns = non-significant).

It has been previously shown that SOD1 variants expressed in cultured cells have different propensities to aggregate (Turner et al., 2005; Prudencio et al., 2009). This variation in aggregation may offer an explanation to the large disparity in average length of disease duration between SOD1 mutations. We first sought to determine the aggregation propensities of our chosen SOD1 variants as EGFP fusion proteins in cultured cells using microscopy. To this end, mouse neuroblastoma/motor neuron hybrid cells (NSC-34) (Cashman et al., 1992) were transfected with SOD1-EGFP constructs and imaged 48 h post-transfection and the number of transfected cells that formed inclusions counted. We chose to work with variants SOD1^WT^, SOD1^G37R^, SOD1^H46R^, SOD1^D90A^, SOD1^G93A^, and SOD1^V148G^ owing to their variable patient survival time (SOD1^G37R^ = ~17, SOD1^H46R^ = ~17.6, SOD1^D90A^ ~8, SOD1^G93A^ ~3.1, SOD1^V148G^ = ~2.1 years) (Wang et al., 2008). We observed that variants, SOD1^G37R^, SOD1^G93A^, and SOD1^V148G^ had significantly increased populations of transfected cells containing inclusions when compared to SOD1^WT^ (Figures 1A,B). In contrast, there was no significant difference between SOD1^WT^ and the variants SOD1^H46R^ and SOD1^D90A^ (Figure 1B). The flow cytometry based FloIT assay showed results similar to manual counting (Figure 1C). While the FloIT technique finds 2–3 times more inclusions per 100 cells, this is due to the fact that multiple inclusions can occur in a single cell (Whiten et al., 2016). Regardless, the outcome from manual counting and FloIT analysis demonstrate that SOD1^G37R^, SOD1^G93A^, and SOD1^V148G^ aggregate significantly more in cells that the other variants tested. We next examined the effects of overexpression of SOD1-EGFP constructs on cell survival through time using real-time imaging. The number of surviving cells was followed and plotted relative to SOD1^WT^ survival (Figure 1D). As previously observed (Turner et al., 2005), the survival of cells expressing SOD1 variants correlated with the number of cells with aggregates (Figure 1E). That is the variants SOD1^G37R^, SOD1^G93A^, and SOD1^V148G^ had significant levels of cell death, while SOD1^H46R^ had an intermediate level of cell death and SOD1^D90A^ was no different to SOD1^WT^. This suggests that the cellular aggregation propensity of the variant predicts cell death in NSC-34 cells.

### Aggregation propensity of recombinant SOD1 does not always predict its cellular aggregation

**Figure 2:**
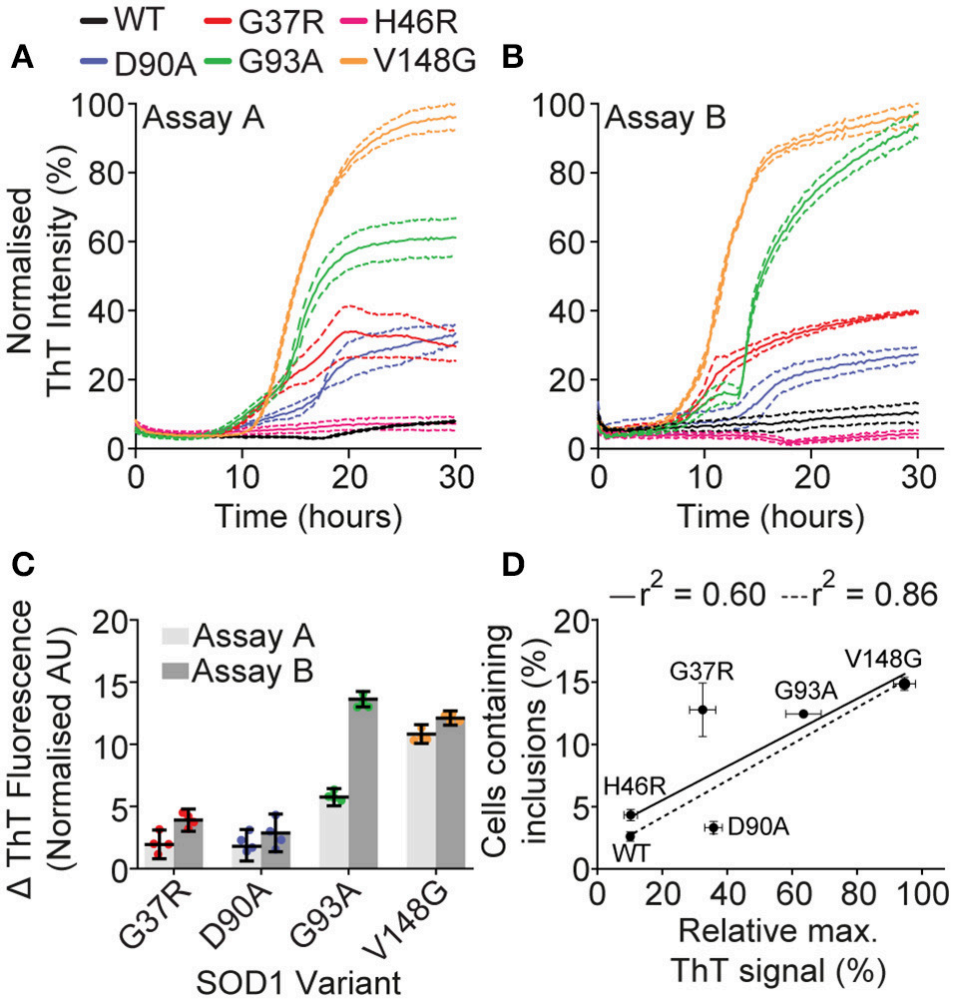
**Isolated recombinant SOD1 aggregation**. Aggregation of purified recombinant SOD1 as promoted with 20 mM DTT 5 mM EDTA in 1 × PBS, with shaking at 37°C, and was monitored by ThT fluorescence. **(A,B)** Plate-based aggregation assay of recombinant SOD1 variants showing the differential aggregation profiles of each variant where each panel represents a separate assay under the same conditions. Dotted lines represent SEM of 4 technical replicates. **(C)** Analysis of the fibril elongation rate (measured as ΔThT fluorescence) of assays **(A,B)**. SOD1^WT^ and SOD1^H46R^ are not included due to the sigmoidal fit being below *r*^2^ = 0.75. **(D)** Plotting the percentage of cells with inclusions against the relative maximum ThT signal from assay A gave an *r*^2^ value of 0.60 (solid black line) which was strengthened to 0.86 (dotted line) when SOD1^G37R^ was omitted from analysis.

The variation in cellular aggregation propensity between SOD1 variants could be due to their intrinsic propensity to aggregate, or given that cells actively generate inclusions in a manner thought to represent a quality control compartment (Weisberg et al., 2012; Farrawell et al., 2015), the level of aggregation may represent an ability of a particular variant to evade cellular protein quality control machinery (Yerbury et al., 2013, 2016). To distinguish between these two possibilities, we sought to assess if there was a correlation between aggregation of purified recombinant SOD1 and SOD1-GFP aggregation in cells. Aggregation of recombinant SOD1 was performed as previously described by us and others (Furukawa et al., 2008; Roberts et al., 2013; Yerbury et al., 2013), making use of DTT (to reduce the intramolecular disulfide) and EDTA (to chelate Cu and Zn), as well as shaking, to promote aggregation of the SOD1 variants. Aggregation was measured via Thioflavin T fluorescence, which has been used as a measure of amyloid formation and is thought to fluoresce when it binds to the β-sheet structure characteristic of amyloid. The aggregation propensities differed between SOD1 variants (Figures 2A,B), with SOD1^V148G^, SOD1^G93A^, SOD1^G37R^, and SOD1^D90A^ obeying Boltzmann-sigmoidal kinetics typical of fibrillar aggregation. SOD1 aggregation is noted to be a highly stochastic process, with large variance between technical and biological replicates (Abdolvahabi et al., 2016); however, a comparison of the rates of fibril elongation (measured as change in ThT fluorescence; Figure 2C) between two assays here showed that inter-assay variation was minimal with the exception of SOD1^G93A^. SOD1^WT^, and SOD1^H46R^ did not fit standard fibrillar aggregation kinetics, and as such analysis of the fibril elongation phase was not performed on these variants. Rather, SOD1^WT^ and SOD1^H46R^ did not seem to aggregate at all under our assay conditions (20 mM DTT, 5 mM EDTA 1 × PBS 37°C, shaking agitation) or was not aggregating into fibrils. For the most part, our assays suggested that propensity to aggregate was dependent upon mutation, and when we plotted the relative final fluorescence of each SOD1 variant in the first isolated aggregation assay (Figure 2A) against the inclusion formation data from transfected mammalian cells (Figure 2D) we observed a relatively weak correlation (*r*^2^ = 0.6). However, if we omit SOD1^G37R^ as an outlier, due to its interestingly high propensity to form inclusions in cells comparative to its patient survival time, the correlation is much stronger (*r*^2^ = 0.86). These data taken together suggest that in our case, with the exception of SOD1^G37R^, aggregation propensity of isolated SOD1 can predict its cellular aggregation.

### DTT and EDTA cause changes in SOD1 oligomeric state

**Figure 3:**
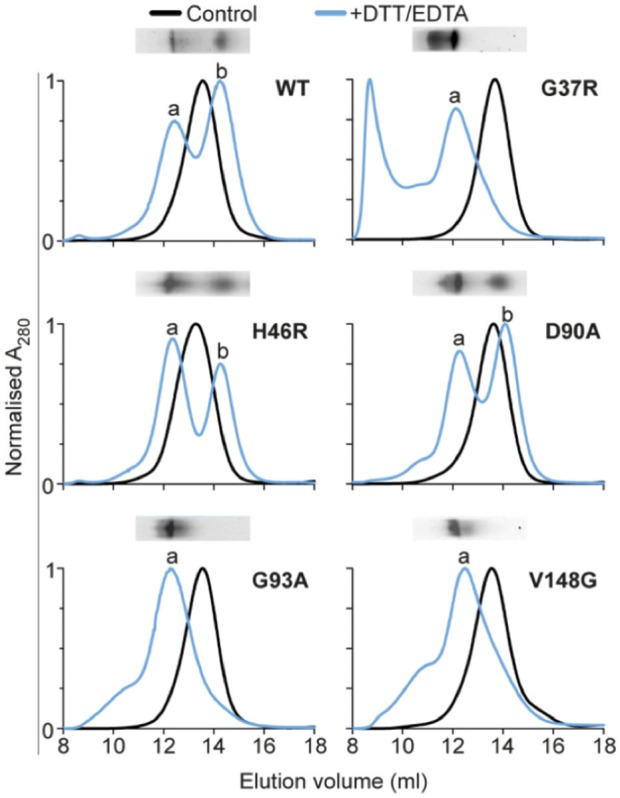
**Analytical size-exclusion chromatography of SOD1 variants**. SOD1 variants were incubated at 37°C, in 1 × PBS, with 20 mM DTT, and 5 mM EDTA, for 2 h. After incubation, samples were concentrated at 4°C to 450 μM in a volume of 100 μl and loaded onto an analytical S-75 column equilibrated in 200 mM NH_4_OAc, and eluted at 0.5 ml/min into 0.5 ml fractions. Black is control native SOD1 which was not incubated in the presence of DTT or EDTA. Blue is SOD1 + DTT + EDTA, which showed different elution profiles for all SOD1 variants, with peaks at ~12 mL being labeled “a” and the peak at ~14 mL being labeled “b.” Inset above each chromatogram is native PAGE of the 2 h incubation time point.

A proposed common characteristic of SOD1 variants is dimer destabilization and dissociation into monomers, which can subsequently lead to the formation of higher order aggregates *in vitro* (Nordlund and Oliveberg, 2008). Indeed, antibodies detecting epitopes exposed only in the misfolded SOD1 monomer show immunoreactivity in motor neurons from human tissue as well as transgenic rat and mouse (SOD1^A4V^, SOD1^G93A^, and SOD1^G37R^) models of disease (Rakhit et al., 2007). To determine if propensity to dissociate to monomer was related to cellular aggregation we used the same destabilizing conditions used above (30 μM SOD1 20 mM DTT, 5 mM EDTA in 1 × PBS) but examined oligomeric state after 2 h (i.e., prior to insoluble aggregates being formed). Native PAGE of our variants at a concentration of 15 μM resolved SOD1^WT^, SOD1^H46R^, and SOD1^D90A^ into clear monomer and dimer populations (Figure 3). Interestingly, we observed no separate monomer or dimer populations for SOD1^G37R^, SOD1^G93A^, or SOD1^V148G^. Rather, the bands seem to indicate that a large proportion of the protein remained dimeric under aggregation inducing conditons. In particular, SOD1^G37R^ showed a substantial difference compared to SOD1^WT^ as there was a large proportion of protein that had a lower mobility when compared to the dimer band, suggesting the presence of larger oligomeric species. Native PAGE has been used previously to investigate the electrophoretic mobility of SOD1 variants following disulfide reduction (Tiwari and Hayward, 2003), where it was proposed that increased mobility was most likely a result of metal loss, and retarded mobility was most likely a result of a conformational change in the protein structure. We next used analytical SEC after incubation in DTT and EDTA, in order to further investigate oligomeric distribution. We observed that the elution profiles of the SOD1 variants corresponded closely to the mobility observed for each variant in the native PAGE assay (Figure 3), suggesting similar changes were detected by each technique. The dimer and monomer populations were, again, apparent for SOD1^WT^, SOD1^H46R^, and SOD1^D90A^, with the dimer eluting at ~12.5 mL, and the monomer at ~14.3 mL. However, the elution profile of the DTT/EDTA-treated SOD1 dimer was distinct to the primarily dimeric non-treated controls (~13.6 mL), indicating a change in the hydrodynamic volume of the SOD1 dimer. This shift to a lower elution volume was observed for all SOD1 variants, with SOD1^G37R^, SOD1^G93A^, and SOD1^V148G^ having major elution peaks at ~12.5 ml, but no peak at ~14.3 mL in contrast to SOD1^WT^, indicating a lack of monomer. Interestingly, SOD1^G37R^ showed substantial protein at the void volume indicating the presence of oligomeric species with masses greater than or equal to ~100 kDa (S-75 10/300 GL column). SOD1^G93A^, SOD1^V148G^, and to a lesser extent SOD1^D90A^ showed the presence of oligomeric species with peak shoulders ranging from ~9 to 11 mL. The analytical SEC, in conjunction with the native PAGE analysis, suggest that after a 2 h treatment with DTT and EDTA, SOD1 undergoes a conformational change that affects the hydrodynamic volume and the packing of the protein. It is well known that changes to the structure of molecules can make SEC data ambiguous (Hong et al., 2012), potentially making the interpretation of these data complicated. Due to this, native mass spectrometry analysis was performed on fractions eluted from analytical SEC assays.

### Putative SOD1 dimer from SEC is unfolded monomer

**Figure 4:**
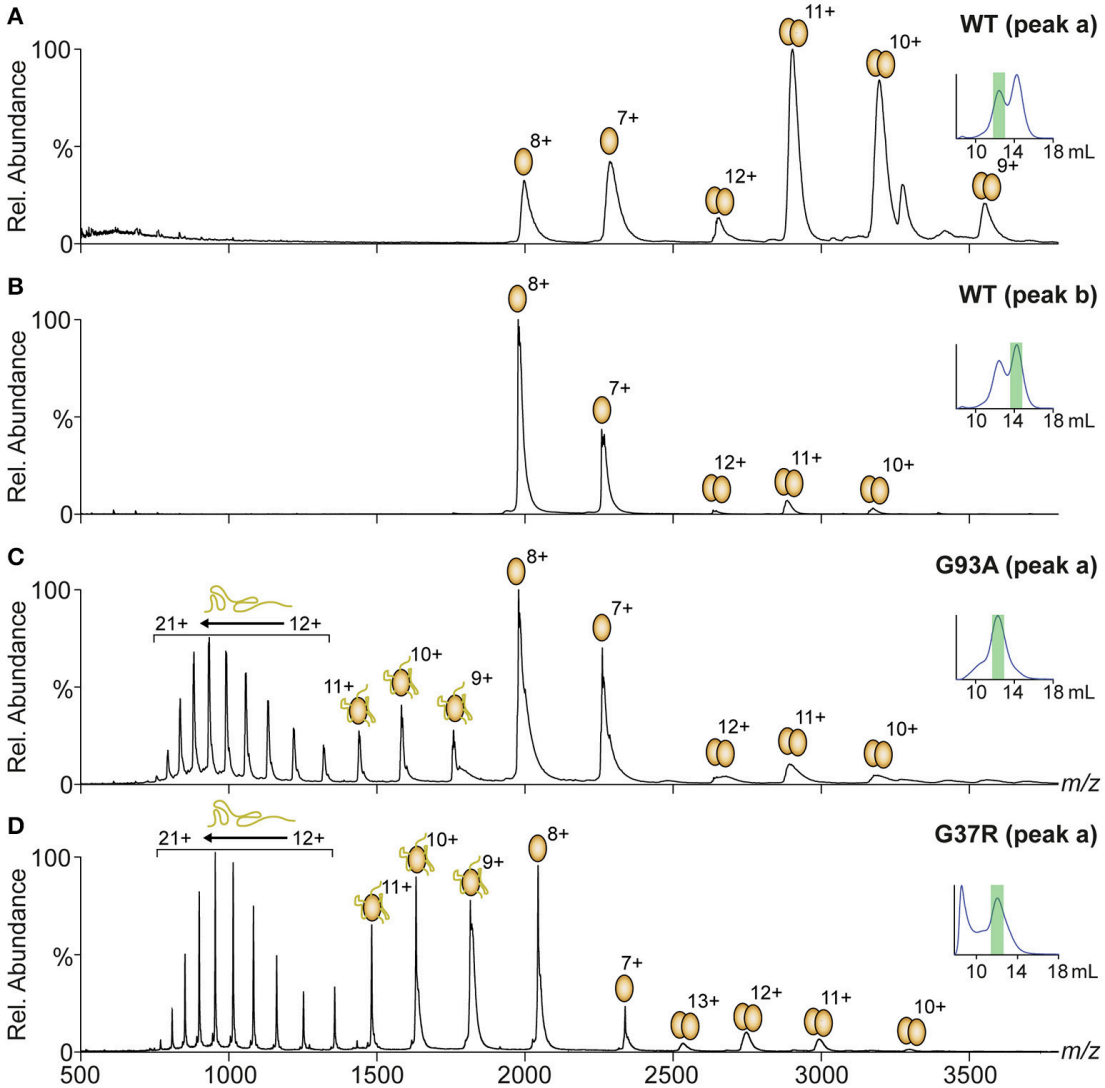
**Native mass spectrometry of peaks from analytical SEC**. Fractions from under peak apexes of DTT/EDTA-treated SOD1 were subject to mass spectrometry under gentle conditions optimized to maintain a “solution-like” conformation. Oligomeric and conformational state is represented by ellipsoids and lines above peaks with charge states marked. **(A)** Spectra of peak “a” from SOD1^WT^ showing a primarily dimeric conformation similar to what is observed in native SOD1 (McAlary et al., 2013). **(B)** Spectra of peak ‘b’ from SOD1^WT^ showing a primarily monomeric conformation at charge states similar to native monomeric SOD1^WT^ (McAlary et al., 2013). **(C)** Spectra of peak “a” from SOD1^G93A^ showing a primarily monomeric distribution with peaks corresponding to monomeric SOD1 with substantially higher charge states than what was observed in SOD1^WT^. **(D)** Spectra of peak′a′ from SOD1^G37R^ showing a similar oligomeric and conformational distribution to SOD1^G93A^ with greater abundance of highly charged (9–21+) monomer.

The native PAGE and analytical SEC data indicated the variable presence of SOD1 dimer and monomer populations depending on the variant. In addition, a potential change in dimer conformation was observed as the dimer peak had a lower elution volume after treatment. Native mass spectrometry (MS) can be used to simultaneously investigate the suite of potential species in this dynamic system, giving high-resolution mass data that can also be used to determine modification states (disulfide status, metal binding, oxidative modification). MS was used to analyse the main peaks eluting from the analytical SEC from Figure 3 (MS analysis in Figure 4), finding that in the case of SOD1^WT^, (Figures 4A,B) the oligomeric distribution in the mass spectra matched the oligomeric state determined from the SEC (i.e., the peak at 12.5 mL was predominantly dimer and the peak at 14.3 mL predominantly monomer). The charge state distributions of both monomer and dimer in this assay were similar to that observed previously for native SOD1 variants (dimer 9 – 12+, monomer 7 – 8+) (McAlary et al., 2013). Analytical SEC of SOD1^G93A^ resulted in only one major peak from DTT/EDTA treatment at 12.5 mL (peak a), consistent with the elution of the DTT/EDTA-treated SOD1^WT^ dimer peak (Figure 3). However, when this was analyzed via MS, it was observed that a substantially higher abundance of monomer ranging from charge states 7+ to 21+ was present (Figure 4C). This broad monomeric charge state distribution was also observed in the ~12 mL peak for SOD1^G37R^ (Figure 4D, peak a), the ~14.2 mL peak for SOD1^D90A^ (Supplementary Figure 1A), and the ~12.1 mL peak for SOD1^V148G^ (Supplementary Figure 1B), although each variant comprised different relative abundances of the highly charged monomer ions (9 – 21+). The charge state distribution of proteins in mass spectrometry is dependent upon conformation (Prakash and Mazumdar, 2005), and the presence of these highly charged monomeric ions is indicative of a variably unfolded protein in the solution phase, due to greater access of solvent to chargeable sites during ionization. SOD1 contains 23 basic amino acids (His = 8, Lys = 11, Arg = 4), and when recombinantly expressed and purified from *E*. *coli*, is methionine cleaved but not acetylated at its N-terminus (Leinweber et al., 2004), meaning it can theoretically carry 23 positive charges if fully unfolded. Our data shows a maximum of 21 charges applied to SOD1^G93A^ (Figure 4), and even in the case of SOD1^G37R^ (Figure 4), which has 24 chargeable sites, we only observed a maximum of 21 charges, suggesting that 2 (3 for SOD1^G37R^) of the basic amino acids are masked from protonation. Taken together we conclude that the monomer ions ranging from 7 to 11+ have maintained some structure after DTT/EDTA treatment, and that 12 – 21+ ions are representative of a highly unfolded monomer.

### SOD1^G37R^ has a high propensity to form oligomeric aggregates

**Figure 5:**
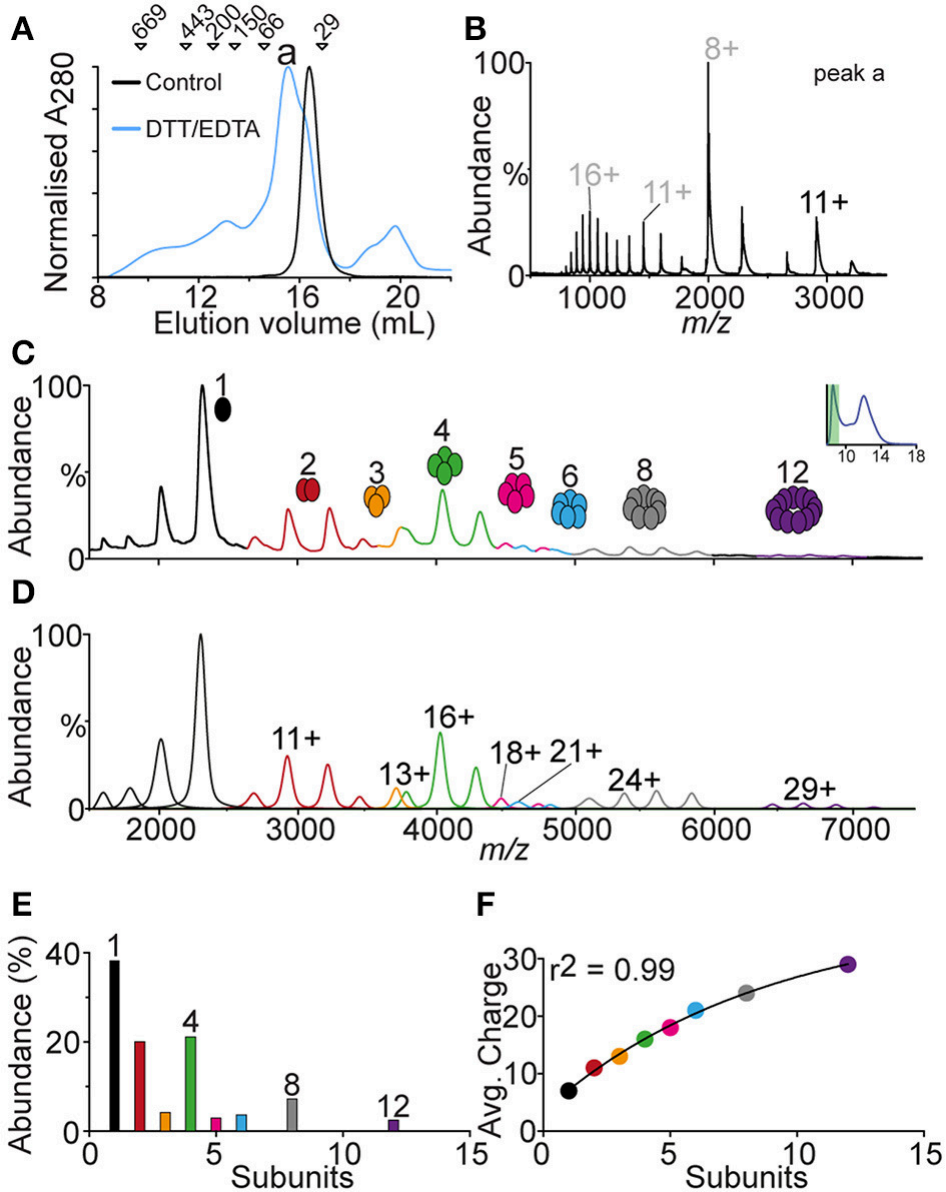
**Analysis of the void volume peak from DTT/EDTA-treated SOD1^G37R^**. Owing to the observation that a large portion of SOD1^G37R^ eluted in the void volume, this fraction was analyzed with native MS. **(A)** S-200 column chromatogram of native SOD1^G37R^ (black) and DTT/EDTA-treated SOD1^G37R^ (blue) showing differences in elution profile where DTT/EDTA-treated is eluting at lower volumes showing a broad oligomeric distribution. The main peak is labeled “a” and markers above the chromatogram represent molecular weight standards. **(B)** Spectra of peak “a” from the S-200 chromatogram showing a similar distribution of SOD1^G37R^ to that seen previously in the S-75 column analysis. **(C)** Spectra of the void volume peak from the SOD1^G37R^ S-75 column analysis showing a broad oligomeric distribution of SOD1^G37R^ ranging from 1 to 12 subunits. **(D)** Theoretical spectra and deconvolution of the mass spectra observed in **(C)**, showing where peak overlap may occur between oligomers, as well as the major charge state of each oligomer. **(E)** Graphing the relative abundance of each oligomer shows a pattern whereby oligomers are formed as groups of 4 subunits due to the relatively low abundance of 2–3 and 5–7 mers and no 9–11 mers. **(F)** Plotting the average charge of each oligomer against the number of subunits in that oligomer gives are strong *r*^2^ value of 0.99, indicating that the association of these oligomers proceeds in a ordered fashion.

The elution profile of SOD1^G37R^, from analytical SEC, showed a large abundance of protein in the void volume, indicating high molecular weight oligomeric species. In order to identify oligomeric species mass spectrometry was performed on the void volume fraction as well as a second analytical size exclusion analysis with freshly prepared DTT/EDTA treated SOD1^G37R^ using an S-200 column (1.3 MDa exclusion limit). The S-200 SEC of SOD1^G37R^ (Figure 5A) showed a primary elution peak for DTT/EDTA treated SOD1^G37R^ at ~15.6 mL compared to the untreated control SOD1^G37R^ at ~16.5 mL. This lower elution volume was consistent with the peaks observed in the S-75 SEC (Figure 3). The absorbance at 280 nm was broad ranging from 8 to 21 mL, with 8–14 mL corresponding to high molecular weight species, and 18–21 mL likely corresponding to residual DTT from the treatment (MS of this peak showed no protein signal–data not shown). To identify the main oligomeric species of the major peak (Figure 5A, peak a), mass spectrometry was performed on this fraction (Figure 5B), which showed the charge state distribution, of primarily monomeric SOD1^G37R^, similar to that observed from the S-75 SEC (Figure 4). The fractions at lower elution volumes, containing oligomeric SOD1^G37R^, were also subject to MS, however low signal was acquired, most likely due to the very low abundance of the oligomers present. To overcome this, the void volume fraction of the SOD1^G37R^ S-75 SEC analysis was subject to MS. We observed a broad distribution of oligomers ranging from 1 to 12 subunits (Figure 5C), with the monomer being the most abundant species, indicating that the oligomers eluted from the column were highly labile. Generation of a theoretical mass spectrum containing the potentially identified species allowed for the correct assignment of each ion species (Figure 5D). We plotted the abundance of each oligomer to determine if there was a pattern of oligomerization, and found that the large oligomers were assembling in patterns of 4 subunits, as shown by the abundance of oligomers composed of 4, 8, and 12 subunits, and the absence of oligomers made from 7, 9, 10, or 11 subunits (Figure 5E). Since the monomer and dimer ions detected from this fraction were showing a charge state distribution typical for SOD1 (monomer = 7−8+, dimer = 9–12+), we plotted the average charge of each oligomeric state against the number of subunits in that oligomer, and found that the charge added per subunit addition was consistent at ~2 charges per subunit added to the complex, indicating that the oligomers were forming in an ordered fashion (Figure 5F).

### Destabilised monomer abundance predicts cellular aggregation

**Figure 6:**
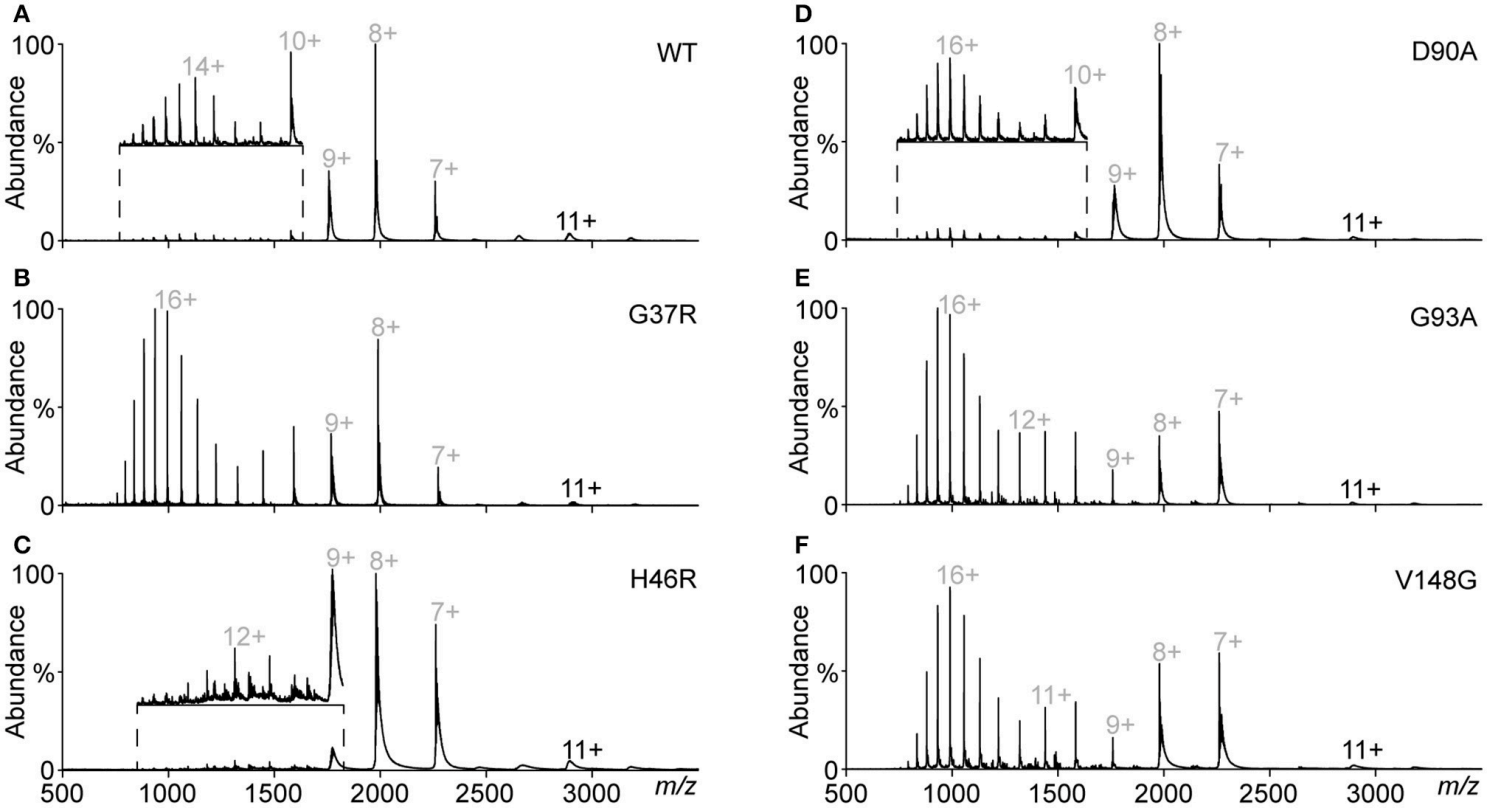
**Mass spectra of pooled DTT/EDTA-treated SOD1 variants from SEC**. Fractions across the elution profile containing SOD1 were pooled, diluted to a concentration of 10 μM, and subject to mass spectrometry. **(A)** SOD1^WT^ showing a high abundance of monomer (gray) compared to dimer (black). Inset is an expansion of the high charged 10 – 21+ monomer ions, normalized to the 10+ ion intensity. **(B)** SOD1^G37R^ spectrum showing that highly charged monomer is the major species present. **(C)** SOD1^H46R^ showing a primarily low charged monomeric distribution. Inset is an expansion of the high charge region normalized to the 9+ ion intensity. **(D)** SOD1^D90A^ showing that the most abundant ion species is the 8+ charge state with low abundance of highly charged monomer. Inset is an expansion of the high charged region, normalized to the intensity of the 16+ ion. **(E)** SOD1^G93A^ showing a substantial abundance of high charged monomer. **(F)** SOD1^V148G^ showing a substantial abundance of high charged monomer.

Given the identification of various states of unfolded monomer from SEC peaks we next wanted to perform a direct comparison of the relative abundances of charge states, and oligomeric distributions for each variant. This was achieved by analyzing the total protein content of each experiment to allow for comparison. Here we observed, that for each SOD1 variant after DTT and EDTA treatment the major species was monomeric, ranging from charges of 7+ to 21+ (Figure 6), as previously observed (Figures 4, 5). The dimer abundance was substantially decreased in all cases, compared to previous spectra that were acquired from peak a in analytical SEC. This indicates that the pooling and dilution of the samples from analytical SEC resulted in dissociation of the dimer. In the case of SOD1^G37R^ (Figure 6B), there was no evidence of any oligomeric species, even though fractions thought to contain them were included into the pooled sample. This is most likely due to a combination of the biases involved in mass spectrometry based analyses, such as this, where system parameters determine sensitivity at specific mass ranges, and the previously observed labile nature of the oligomers. In contrast to previously acquired spectra from fractions corresponding to peaks in the analytical SEC (Figures 4, 5), it was observed that highly charged monomer was present for all SOD1 variants, although at different abundances for each SOD1 variant. SOD^WT^, SOD1^H46R^, and SOD1^D90A^ (Figures 6A,C,D, respectively) showed relatively low abundances of highly charged SOD1 monomer, instead the major ion species was the monomeric 8+ charge state. SOD1^G37R^, SOD1^G93A^, and SOD1^V148G^ (Figures 6B,E,F, respectively) had considerably increased relative abundances of highly charged monomeric species, in comparison to the typical 7+ and 8+ monomeric charge states.

**Figure 7:**
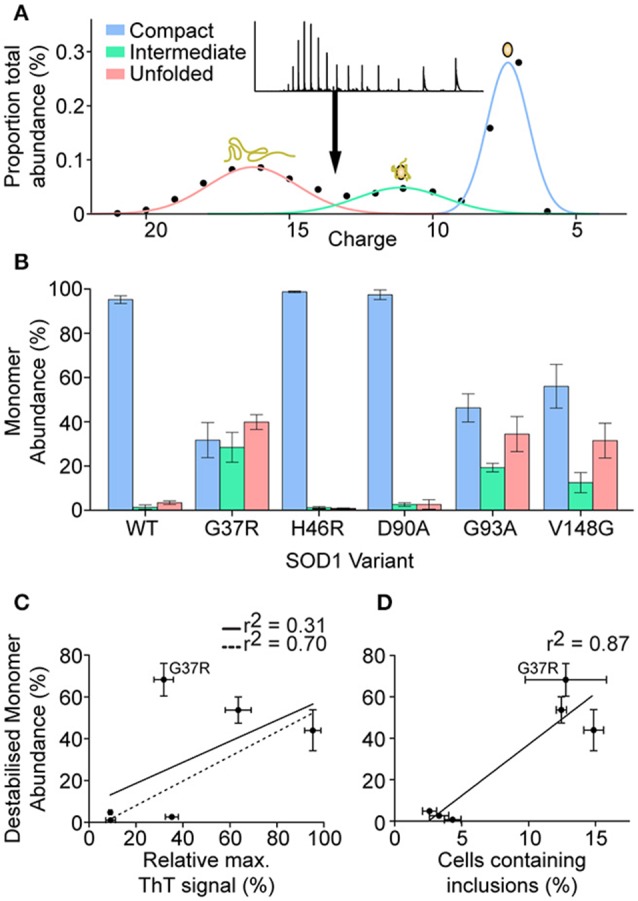
**Determination of the abundance of monomeric conformations detected by mass spectrometry. (A)** Plotting the abundance of each charge state allowed for the determination of 3 Gaussian distributions that correspond to increasing charge states and decreasing peak width, indicative of 3 conformations (compact = blue, intermediate = green, unfolded = red). **(B)** The abundance of each specific conformation for each SOD1 variant shows the monomeric distribution where SOD1^WT^, SOD1^H46R^, and SOD1^D90A^ present a primarily compact monomeric distribution, compared to the other variants which showed high abundances of intermediate and unfolded monomer conformations. Error bars represent SD of the mean from 3 separate DTT/EDTA treatments of 3 separate nano-electrosprays. **(C)** Plotting the percentage abundance of destabilized monomer (unfolded and intermediate conformation) against the relative ThT intensity from aggregation assay A (Figure 2) showed a weak correlation (*r*^2^ = 0.31), however omission of SOD1^G37R^ from the linear fit gave a much stronger correlation (*r*^2^ = 0.70). **(D)** Plotting the percentage abundance of destabilized monomer against the percentage of cells containing inclusions after transfection showed a strong correlation (*r*^2^ = 0.87). Error bars represent SD of the mean from at least 3 separate experiments.

Following the analysis and identification of protein species evident from a close inspection of different monomer ions, it was determined that there were three main monomeric conformations that were being detected by MS. These conformations were termed compact, intermediate, and unfolded due to the major species across the monomeric ions being reduced apo-SOD1 for all variants i.e., no holo-SOD1 was observed. It was also based on the Gaussian distribution of peaks (Figure 7A) where we determined the relative abundance of each peak and plotted the abundance of each charge state. From these Gaussian fits it was possible to determine the relative abundance of each conformation for all SOD1 variants (Figure 7B). We found that SOD1^WT^, SOD1^H46R^, and SOD1^D90A^ monomers primarily inhabited the compact conformation, with much lower abundances of intermediate and unfolded conformations. SOD1^G37R^, SOD1^G93A^, and SOD1^V148G^ had far higher abundances of intermediate and unfolded monomer, compared to SOD1^WT^, and out of the three variants, only SOD1^G37R^ showed a higher abundance of unfolded monomer compared to compact monomer. Since SOD1 aggregation has been previously proposed to be dependent upon the amount of unfolded/destabilized protein (Byrstrom et al., 2010), the relative ThT intensity from previous aggregation data (Figure 2A) was plotted against the percentage of monomer that was destabilzed (sum of intermediate and unfolded abundances) (Figure 7C). The correlation was relatively weak (*r*^2^ = 0.31), however when SOD1^G37R^ was omitted from the linear fit, the correlation became much stronger (*r*^2^ = 0.70). SOD1^G37R^ is a curious outlier as it is defined as “wild-type like” due to its structural similarities with SOD1^WT^ (Hart et al., 1998) and has a long variable disease duration of ~17 years (Wang et al., 2008), yet in our assays presents severe structural destabilization on similar levels as the aggressive SOD1^G93A^ and SOD1^V148G^ variants (~2.2 and ~2.3 years patient survival, respectively). Given that aggregation propensity did not very well predict cellular aggregation we next asked whether the relative abundance of destabilized monomer correlated with cellular aggregation (Figure 7D). Here the correlation was very strong (*r*^2^ = 0.87) and as such it seems that the main predictor of cellular aggregation is the propensity of the SOD1 variants to unfold as previously suggested (Nordlund and Oliveberg, 2008).

### Unfolded SOD1^G37R^ does not template on to SOD1^G93A^ seeds

**Figure 8:**
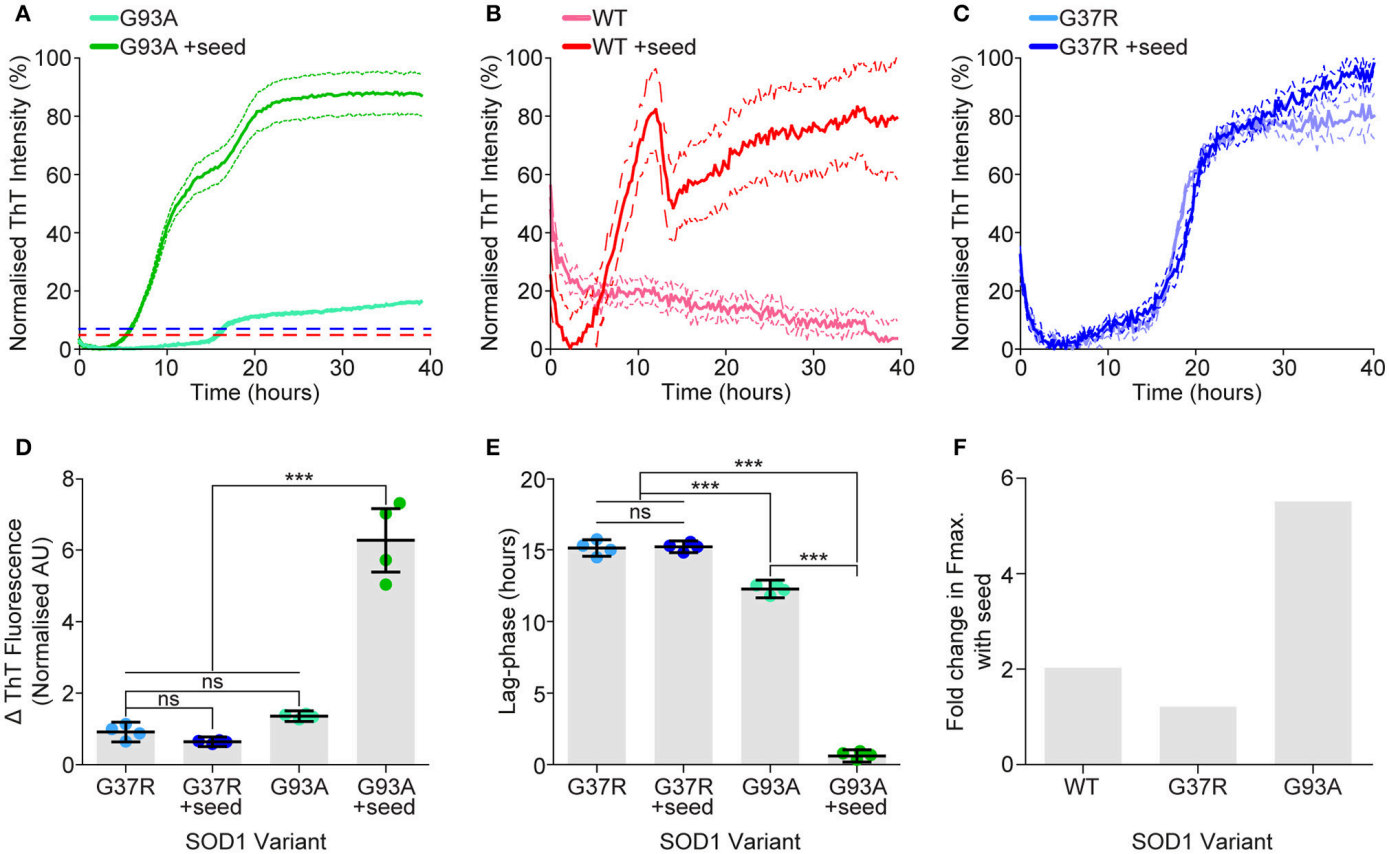
**SOD1^G37R^ is resistant to seeding by preformed SOD1^G93A^ aggregates**. Aggregation of SOD1 was promoted with DTT, EDTA, and shaking, with the formation of fibrillar structures being monitored by ThT fluorescence. Preformed SOD1^G93A^ aggregates were added (or not) at a final amount of 0.3% total protein. **(A)** Aggregation assay of SOD1^G93A^ normalized to the maximum signal showing the ThT fluorescence over time for each sample. Dotted lines represent the maximum fluorescence of SOD1^WT^ (red) and SOD1^G37R^ (blue) assays shown in panels B and C, respectively. **(B)** Aggregation assay of SOD1^WT^ with and without seed, normalized to the maximum fluorescent signal. **(C)** Aggregation assay of SOD1^G37R^ with and without seed, normalized to the maximum fluorescence signal **(D)** Kinetic analysis gave the fibril elongation rate (measured as change in ThT fluorescence), showing that seeded SOD1^G93A^ had a significantly increased fibril elongation rate compared to other treatments. SOD1WT is not shown here due to it not aggregating without seed. **(E)** Kinetic analysis gave the lag phase of aggregation, showing that addition of seed significantly decreased the lag phase of SOD1^G93A^ but not SOD1^G37R^. **(F)** Comparing the fold increase in the max fluorescence (Fmax) values when aggregation was seeded for each variant, showing that SOD1^G93A^ and SOD1^WT^ had greater increases compared to SOD1^G37R^. Each assay presented here is representative of 2 separate assays and were performed in the same microplate where error bars represent SEM of 4 technical replicates. Statistical significance was determined using a one-way ANOVA with a Tukey's post-test (^***^P <0.001).

While we observe a strong correlation between propensity to unfold, cellular aggregation and resultant cell loss, translating this to human disease severity is limited by the fact that one of our variants SOD1^G37R^ has a high propensity to aggregate and is toxic in cells but has a long mean disease duration of 17 years. The concepts of aggregate strains and prion-like seeding have recently become prominent within the study of neurodegenerative disease, especially relating to ALS (Grad et al., 2014; Bergh et al., 2015; Lang et al., 2015; Bidhendi et al., 2016). Owing to the fact that spinal cord material from SOD1^G37R^ mice were unable to propagate disease in SOD1^G85R^ mice while SOD1^G93A^ material was (Ayers et al., 2014), we next performed an experiment to elucidate if this unfolded species could template onto other preformed SOD1 aggregates. SOD1^G93A^ was chosen to be the ‘seed’ variant, as we had previously shown it could seed SOD1^WT^ aggregation (Grad et al., 2014), in this experiment a minimal amount of preformed aggregate seed (seed was 0.3% total protein by mass) was added to the assay. Remarkably, it was observed that SOD1^G37R^ did not respond to the addition of seed, whereas SOD1^WT^ showed an appreciable response (Figures 8B,D) and SOD1^G93A^ showed significantly altered aggregation kinetics (Figures 8A,D). SOD1^G37R^ showed no difference in either fibril elongation rate (measured as change in ThT fluorescence) (Figures 8C,D), or lag phase (Figure 8D), indicating that the addition of the seed was not affecting either the nucleation time, or the generation of fibrillar structures. The kinetics of SOD1^WT^ were difficult to determine due to a weak sigmoidal fit, however it was clear from the assay (Figure 8A) that the addition of SOD1^G93A^ seed increased aggregation. Addition of seed to SOD1^G93A^ aggregation significantly increased the elongation rate (Figure 8D) compared to no seed, and also significantly decreased the lag phase (Figure 8E), indicating that the addition of seed was pushing the aggregation reaction toward fibril elongation, overcoming the need to form aggregate nuclei. Next we compared the fold increase in the mean maximum ThT fluorescence between variants (Figure 8F) after seeding and found that SOD1^G37R^ had only a slight increase in max fluorescence, compared to SOD1^WT^ which was higher than SOD1^G37R^ and SOD1^G93A^ which was substantially higher than both other variants. Taken together, this work suggests that SOD1^G37R^ may be aggregating as a different strain that is dependent upon the substitution of arginine at position-37.

## Discussion

In this work we have shown that the reduction and chelation of SOD1 variants induces unfolding into multiple conformational states, and that the major predictor for cellular aggregation of SOD1 variants is the abundance of destabilized SOD1 states. It is well known that disruption of the metal ions and the intramolecular disulfide has severe effects upon SOD1 structural stability (Hayward et al., 2002; Tiwari and Hayward, 2003; Lynch et al., 2004; Banci et al., 2009; Svensson et al., 2010). Indeed, in the native conformation (metal bound, disulfide bonded form) most ALS-associated SOD1 variants have high melting temperatures, however, when reduced and metal-free, some variants, such as SOD1^G93A^, melt below physiological temperature (Furukawa and O'Halloran, 2005; Rodriguez et al., 2005). Taking this into account, it is clear that the conformational distribution we observed in MS assays is a result of the lower thermal stabilities of the SOD1 variants when in their reduced apo-form, which was induced via incubation in DTT and EDTA at physiological temperature. Take SOD1^G93A^ for example, which in its reduced apo-form, has a reported melting temperature of 31.2 ± 0.3°C (Furukawa and O'Halloran, 2005). We observed that SOD1^G93A^ was significantly destabilized following DTT/EDTA treatment at physiological temperature, in agreement with this previous work. The most interesting aspect of the MS-based assay however, was that it was able to further separate the products of destabilization, determining the existence and relative abundance, of several conformational states. Previously published thermal stability data has not detected these states (Furukawa and O'Halloran, 2005; Rodriguez et al., 2005), most likely due to the low resolution provided by the technique, or that these conformations are in a constant state of flux. Higher resolution analyses of disordered SOD1 have been conducted using NMR, where reduced apo-SOD1^G93A^ was found to be highly unstructured, with little propensity to form secondary structure (Luchinat et al., 2014), and reduced apo-SOD1^WT^ was found to assume a monomeric native-like β-barrel structure with disordered loops (Sekhar et al., 2015). This model of reduced apo-SOD1^WT^ matches the analysis presented here where the relatively low charge state distribution is indicative of a more compact monomeric species, close to what we observed for disulfide oxidized holo-SOD1^WT^.

Likewise, other work has considered reduced apo-SOD1^G93A^ to be representative of several other SOD1 variants, including SOD1^G37R^, which is in partial agreement with our results (Luchinat et al., 2014). SOD1^G37R^ was the most destabilized variant observed, with SOD1^G93A^ a close second. In-cell NMR spectroscopy revealed that the levels of unstructured reduced apo-SOD1^G37R^ and reduced apo-SOD1^G93A^ were similar when overexpressed in mammalian cells, failing to detect any tertiary structure (Luchinat et al., 2014). In contrast, the MS-based assay performed here detected reduced apo-SOD1 species inhabiting quaternary, tertiary, and unstructured states, as determined by IM-MS, across all of our SOD1 variants. The discrepancy is potentially due to the experimental design, i.e., the work present here has investigated SOD1 that has been unfolded from its native conformation, whereas Luchinat et al. (2014) and colleagues have investigated SOD1 structures that may not yet be folded. Regardless of the order, the removal of post-translational modifications from SOD1 is widely thought to result in a product that is similar to the immature polypeptide that can take part in aggregation.

The isolated protein and cell-based aggregation assays showed a clear difference between specific SOD1 variant aggregation propensities. For the most part, aggregation in the cellular context correlated with aggregation of isolated protein, with the exception of SOD1^G37R^. Ivanova et al. (2014) determined that the SOD1 sequence segment ^30^KVWGSIKGL^38^ was a fibril forming segment but the same peptide containing the SOD1^G37R^ mutation did not form fibrillar structures, suggesting that the SOD1^G37R^ mutation may lead to aggregation through other fibril forming segments or to amorphous aggregation. SOD1^G37R^ was a consistent outlier throughout this work, and even more so when taking into account the interesting correlation between patient survival time and SOD1 mutation, where in general SOD1 variants more prone to aggregation are correlated with a more rapid disease progression (Wang et al., 2008). Given the association with a long disease course, one might expect SOD1^G37R^ to have a low aggregation propensity in cells, as well as a low isolated protein aggregation propensity. However, its in-cell aggregation was significantly increased compared to SOD1^WT^, similar to that observed for SOD1^G93A^ and SOD1^V148G^. A potential explanation for this is the difference between the intracellular environment compared to that of an isolated protein assay. The isolated protein assay contains only pure SOD1 protein at a relatively low concentration (uncrowded) of 30 μM dimer, and is optimized to produce fibrillar aggregates at a rapid rate with vigorous shaking. The cytoplasm contains an abundance of protein and other macromolecules in high concentration (~400 mg/mL), resulting in a crowded environment (Fulton, 1982; Zeskind et al., 2007). This crowding effect, along with other cellular processes, are likely changing the dynamics of SOD1 aggregation, when compared to isolated protein. Indeed, transfection of cells typically results in variable expression levels of the protein of interest, across the population of cells that have taken up the vector. In the case presented here, the highest expressing cells are the ones most likely to develop inclusions, consistent with the idea that protein concentration is an important factor contributing to aggregation. Inducing high expression levels of SOD1 in the absence of similarly high expression of the copper-chaperone for SOD1 (CCS) is likely to result in a large pool of reduced apo-SOD1 that is more susceptible to aggregation. Recent work has highlighted a faulty interaction between SOD1^A4V^ and human CCS (Wright et al., 2016). This suggests that SOD1 aggregation may result from an overloading of its specific quality control systems in motor neurons. Another explanation is the energy driven aggregation that occurs in cells. Previous work has shown that large inclusions (similar to the inclusions we examine here) are dependent upon microtubule transport (Farrawell et al., 2015) and are not a simply a product of random aggregation. It is possible that aggregation occurs after misfolded SOD1 has been transported and concentrated at the site of an inclusion.

The analytical SEC and MS analysis lends credence to the concept of crowding, where destabilized SOD1 was concentrated prior to SEC (~450 μM), however, the presence of a substantial amount of high molecular weight species was observed only in the case of SOD1^G37R^, indicating a rapid formation of oligomeric species for this variant. These oligomeric species were sensitive to dilution, as evidenced by the most abundant species in our MS analysis of the SOD1^G37R^ void volume being monomeric. This potentially translates into a greater cellular aggregation propensity due to the high concentration of SOD1 with certain cells, but a lower propensity of isolated protein to aggregate due to the comparatively decreased concentration of SOD1 in our assay. Furthermore, there is a possibility that SOD1^G37R^ forms aggregate nuclei rapidly, leading to a decreased pool of available monomer to template onto extending fibrils, although we cannot rule out the possibility of a competing amorphous aggregation pathway for SOD1^G37R^. Interestingly, while in many neurodegenerative diseases soluble hydrophobic oligomeric aggregates are implicated as the most toxic aggregate species (Bolognesi et al., 2010), here we find the opposite; SOD1^G37R^ has the highest propensity to form oligomers, but is the amongst the slowest progressing mutants in humans.

**Figure 9:**
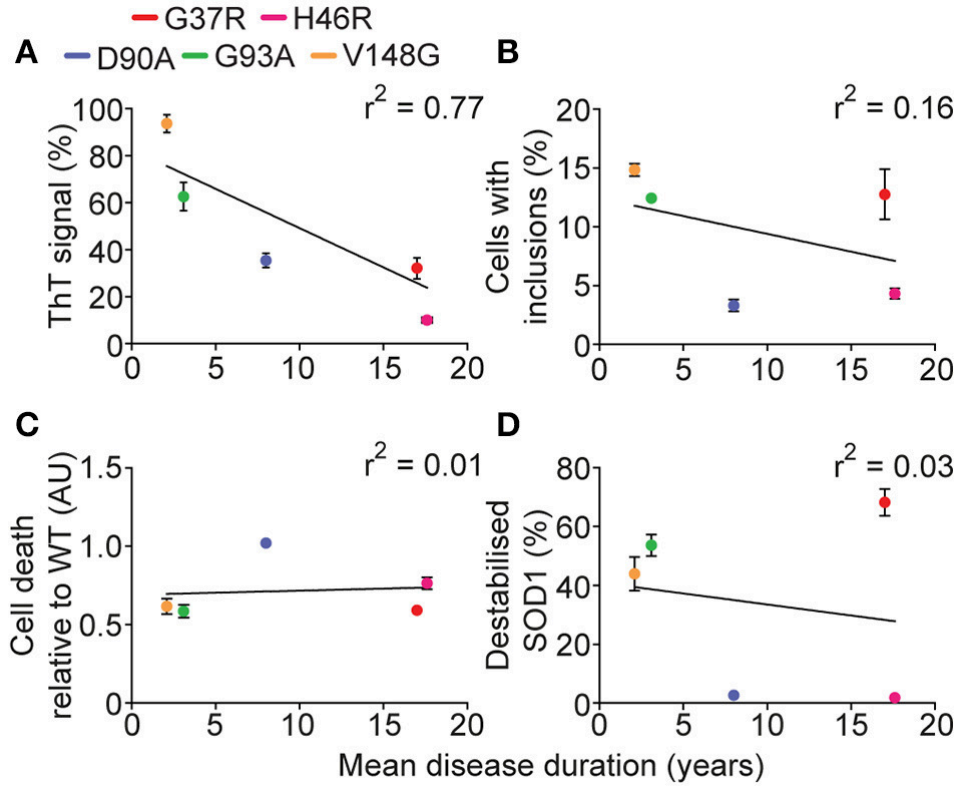
**Correlation of measured variables to human disease duration**. Since sufferers of SOD1-associated fALS cases have variable survival times post-diagnosis, the measured variables in this work were plotted against mean disease duration. **(A)** The relative maximum ThT signal from *in vitro* aggregation assay plotted against disease duration gave a plausible correlation (*r*^2^ = 0.77). **(B)** The percentage of cells containing inclusions 48 h post-transfection plotted against disease duration gave a very weak correlation (*r*^2^ = 0.16). **(C)** Plotting the cell death comparative to SOD1^WT^ 48 h post-transfection gave a very weak correlation (*r*^2^ = 0.01). **(D)** Plotting the relative abundance of unfolded SOD1 measured from MS assays against disease duration gave a very weak correlation (*r*^2^ = 0.03).

Recent work has emphasized the role that different aggregation pathways, leading to the formation of different aggregate strains, may play in the role of amyotrophic lateral sclerosis (Bergh et al., 2015; Lang et al., 2015; Bidhendi et al., 2016). Using binary epitope mapping, Bergh et al. (2015) determined that mice overexpressing human SOD1^D90A^ developed SOD1 aggregates that were found to be competing strains termed strain A and B. Each strain demonstrated differential kinetics and mortality in mice. A follow on from this saw the injection of mice expressing human SOD1^WT^ with either of these strains developing ALS-like symptom at different rates, and also presented different pathophysiology (Bidhendi et al., 2016). The strain hypothesis may explain, at least in part, why different SOD1 mutants present variable disease progression in patients. Indeed, the work presented here indicates that SOD1^G37R^ may be aggregating into a strain distinct from SOD1^G93A^ similar to previous work that suggested SOD1^G37R^ mice spinal cord homogenates could not transmit disease to a SOD1^G85R^ mouse, but that SOD1^G93A^ mice homogenates could transmit disease (Ayers et al., 2014). Given the MS data showed that SOD1^G37R^ had the highest abundance of unfolded monomer, it might be predicted to have substantially altered aggregation dynamics in the presence of preformed aggregate seed (decreased lag phase, increased elongation rate), however this was not the case. Previous work has shown that the SOD1 sequence segment ^30^KVWGSIKGL^38^ is fibril forming (Ivanova et al., 2014), and in conjunction with our observations presented here suggests it is possible that SOD1^G93A^ fibrils are polymerizing based on this segment, and therefore the seeded aggregation of SOD1^G37R^ did not take place due to the arginine substitution at position 37. This combined with the observation of high levels of unfolded SOD1^G37R^ in the cell-free assays would infer SOD1^G37R^ fibril formation is dependent upon other sequence segments, however, we still observe a low *in vitro* aggregation propensity for this SOD1 variant, indicating that the sequence segment ^30^KVWGSIKGL^38^ may indeed be the primary fibril forming segment within SOD1. This SOD1^G37R^ aggregate strain may be, for the most part, benign, explaining the comparatively long disease duration of this SOD1 variant, although further characterization of *in vitro* and *in vivo* SOD1^G37R^ aggregates would need to be performed to confirm this interpretation. Interestingly, of the variables measured here, including *in vitro* aggregation, cellular aggregation, structural destabilization, and cell death, the strongest correlation to human SOD1-associated fALS patient survival was *in vitro* aggregation (Figure 9A). Cellular inclusion formation, cell death, and structural destabilization all had very weak correlations with patient survival (Figures 9B,C,D) again consistent with the idea that a strain of aggregate reactive with ThT might best predict disease severity.

In conclusion, the work presented here underscores the necessity of combining mass analysis techniques to size determination techniques, as the relationship between size and mass may be drastically altered by conformational changes to molecules. Native mass spectrometry is an excellent technique as it provides a means by which to not only separate different conformers, but identify modification states, as we have shown above. The conformers we have identified related to different monomeric conformational states of several ALS-associated SOD1 variants, showing that disulfide reduction and metal chelation resulted in differential conformational states, which correlates with cellular aggregation and toxicity. SOD1^G37R^ presented itself as an outlier when taking into account its high abundance of unfolded protein and oligomeric species, yet a comparatively low *in vitro* aggregation propensity. Seeding SOD1^G37R^ aggregation with preformed SOD1^G93A^ aggregates had no effect on its aggregation kinetics, suggesting that SOD1^G37R^ may aggregate as a different strain and thus potentially explain its slow disease progression. Understanding the interplay between unfolding and subsequent aggregation, as well as the potential for the formation of variable aggregate strains of more SOD1 variants may shed light on the phenomenon of SOD1 variant related differential patient survival times, and may also provide information on the disease causing species in SOD1-associated ALS.

## Author contributions

LM performed experiments, interpreted and analyzed data, as well as wrote the initial manuscript. JA designed experiments, interpreted and analyzed data. JJY designed experiments, interpreted and analyzed data, and wrote and edited the manuscript.

## Funding

JJY is supported by an NHMRC Career Development Fellowship (1084144) and a Dementia Teams Grant (1095215). LM was supported by a University of Wollongong matching scholarship.

### Conflict of interest statement

The authors declare that the research was conducted in the absence of any commercial or financial relationships that could be construed as a potential conflict of interest.
