# Supplementary material for: Susceptibility of Mutant SOD1 to Form a Destabilized Monomer Predicts Cellular Aggregation and Toxicity but Not In vitro Aggregation Propensity
Source: Front Neurosci. 2016 Nov 4;10:499. doi: 10.3389/fnins.2016.00499 (PMC5095133; doi:10.3389/fnins.2016.00499)
Supplement: Supplementary file 1 [file Image1.pdf]

Supplementary Material

Susceptibility of mutant SOD1 to form a destabilized monomer predicts cellular aggregation and toxicity but not *in vitro* aggregation propensity

Luke McAlary, J. Andrew Aquilina, Justin J. Yerbury*

*** Correspondence:** Justin J. Yerbury: jyerbury@uow.edu.au

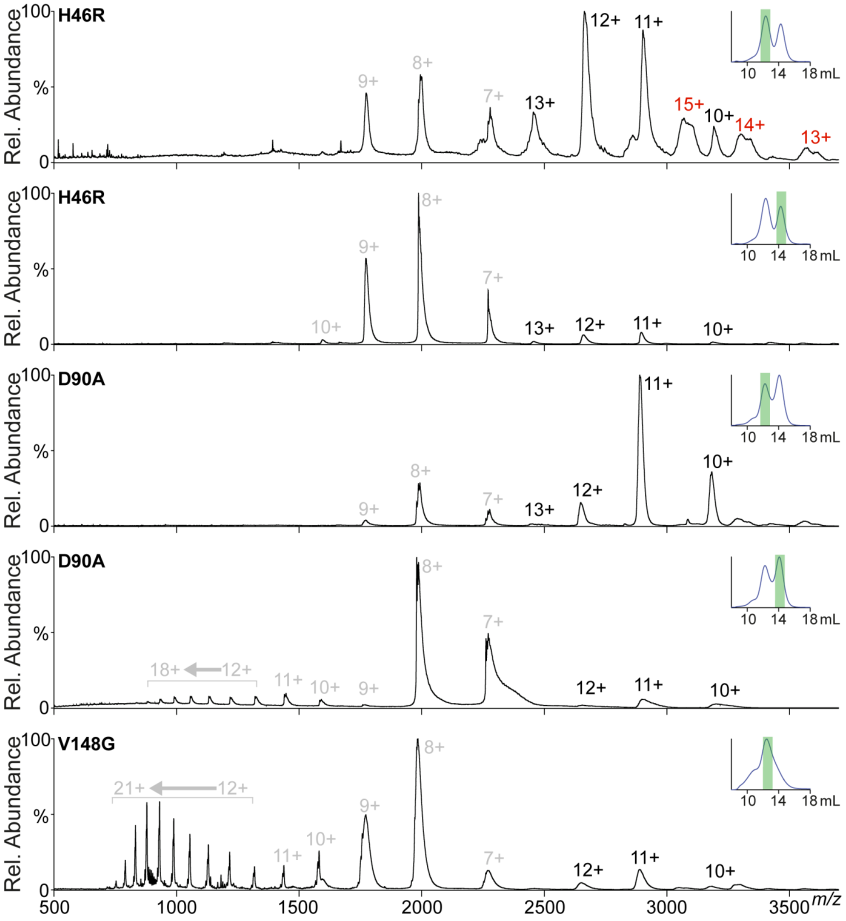

Supplementary Figure 1. Mass spectra of peak apexes from $SOD1^{H46R}$, $SOD1^{D90A}$ and $SOD1^{V148G}$ analytical SEC. Labels are representative of charge state and oligomeric distribution (red = trimer, black = dimer, grey = monomer. i.e. black 12+ = 12+ dimer charge state).
